# Extrahepatic Surgery in Cirrhosis Significantly Increases Portal Pressure in Preclinical Animal Models

**DOI:** 10.3389/fphys.2021.720898

**Published:** 2021-08-20

**Authors:** Johannes Chang, Jonathan Meinke, Moritz Geck, Marc Hebest, Nina Böhling, Ramona Dolscheid-Pommerich, Birgit Stoffel-Wagner, Glen Kristiansen, Marcus Overhaus, Leon O. Peyman, Sabine Klein, Frank E. Uschner, Maximilian J. Brol, Tim O. Vilz, Philipp Lingohr, Jörg C. Kalff, Christian Jansen, Christian P. Strassburg, Sven Wehner, Jonel Trebicka, Michael Praktiknjo

**Affiliations:** ^1^Department of Internal Medicine 1, Center for Cirrhosis and Portal Hypertension Bonn (CCB), University Hospital Bonn, Bonn, Germany; ^2^Department of Clinical Pharmacology, University Hospital Bonn, Bonn, Germany; ^3^Institute of Pathology, University Hospital Bonn, Bonn, Germany; ^4^Department of Visceral Surgery, Malteser Hospital Sankt Hildegardis, Cologne, Germany; ^5^Translational Hepatology, Department of Internal Medicine 1, University of Frankfurt, Frankfurt, Germany; ^6^Department of Surgery, University of Bonn, Bonn, Germany; ^7^European Foundation for the Study of Chronic Liver Failure, Barcelona, Spain

**Keywords:** surgery, acute decompensation, cirrhosis, ACLF, portal pressure, HVPG, intestinal manipulation

## Abstract

**Background:** Liver cirrhosis is a relevant comorbidity with increasing prevalence. Postoperative decompensation and development of complications in patients with cirrhosis remains a frequent clinical problem. Surgery has been discussed as a precipitating event for decompensation and complications of cirrhosis, but the underlying pathomechanisms are still obscure. The aim of this study was to analyze the role of abdominal extrahepatic surgery in cirrhosis on portal pressure and fibrosis in a preclinical model.

**Methods:** Compensated liver cirrhosis was induced using tetrachlormethane (CCL4) inhalation and bile duct ligation (BDL) models in rats, non-cirrhotic portal hypertension by partial portal vein ligation (PPVL). Intestinal manipulation (IM) as a model of extrahepatic abdominal surgery was performed. 2 and 7 days after IM, portal pressure was measured *in-vivo*. Hydroxyproline measurements, Sirius Red staining and qPCR measurements of the liver were performed for evaluation of fibrosis development and hepatic inflammation. Laboratory parameters of liver function in serum were analyzed.

**Results:** Portal pressure was significantly elevated 2 and 7 days after IM in both models of cirrhosis. In the non-cirrhotic model the trend was the same, while not statistically significant. In both cirrhotic models, IM shows strong effects of decompensation, with significant weight loss, elevation of liver enzymes and hypoalbuminemia. 7 days after IM in the BDL group, Sirius red staining and hydroxyproline levels showed significant progression of fibrosis and significantly elevated mRNA levels of hepatic inflammation compared to the respective control group. A progression of fibrosis was not observed in the CCL4 model.

**Conclusion:** In animal models of cirrhosis with continuous liver injury (BDL), IM increases portal pressure, and development of fibrosis. Perioperative portal pressure and hence inflammation processes may be therapeutic targets to prevent post-operative decompensation in cirrhosis.

## Introduction

Liver cirrhosis is the common end-stage of chronic liver diseases. Acute decompensation (AD) such as variceal bleeding, refractory ascites, hepatorenal syndrome, or hepatic encephalopathy can develop and define advanced stages (Angeli et al., 2018). AD may also precipitate acute-on-chronic liver failure (ACLF), a distinct syndrome recently characterized in the CANONIC- and PREDICT-study (Moreau et al., 2013; Gustot et al., 2015; Trebicka et al., 2019, 2020a,b). ACLF is defined by the development of multiorgan failure resulting in high short-term mortality.

Postoperative decompensation of cirrhosis is a well-known but still unsolved problem in surgery. Even though there has been substantial progress in the fields of hepatology and surgery in managing patients with cirrhosis, surgery-associated AD and mortality remains high and correlates with severity of liver disease (Friedman, 2010; de Goede et al., 2012). Recently, the role of surgery as a precipitating event for ACLF development has been characterized, resulting in high rates of ACLF development even after electively performed surgical procedures (Klein et al., 2020; Chang et al., 2021). Therefore, in many hospitals, with the presence of cirrhosis especially in advanced stages is considered a contraindication for all kinds of surgery.

Clinically significant portal hypertension has been associated with increased numbers of episodes of acute decompensation after hepatic surgery (Bruix et al., 1996). In a recent prospective study, hepatic venous pressure gradient (HVPG) has also been described as a predictor for mortality after extrahepatic surgery, indicating that optimization of portal hypertension might be the key to improve postoperative outcome (Reverter et al., 2019). However, data about underlying mechanisms of post-operative decompensation and characterization of portal pressure in the pre- and post-operative period are at best scarce and thus need to be studied more to shed light on the pathophysiology involved in the post-operative development of AD and ACLF.

In this context, preclinical models to characterize proinflammatory downstream signaling and portal hemodynamics that help to understand the pathophysiology of post-operative decompensation of cirrhosis are needed. This study aimed to establish a preclinical model of extrahepatic abdominal surgery in animal models of portal hypertension and to study consecutive changes of portal pressure and liver fibrosis.

## Materials and Methods

### Animal Experiments

Specific pathogen-free male Sprague Dawley rats were used for this study. Animals were acquired from Charles-River (Sulzfeld, Germany) and maintained in the animal facility at the University Clinic of Bonn, Department for Experimental Therapy in individually ventilated cages with a 12:12-h day-night cycle at 22 °C. Water and chow were provided *ad libitum*. Animal studies were performed in accordance with the German Animal Welfare Act and standard operation procedures of the Laboratory of Liver Fibrosis and Portal Hypertension and the animal care facility. Studies were approved by the Landesamt für Natur, Umwelt und Verbraucherschutz Nordrhein-Westfalen (LANUV, Reference: 81-02-04.2018.A348). Animals were sufficiently handled before all operations and received sufficient pain medication after all operations. When reaching human endpoint the experiment was stopped and animals were euthanized.

### Establishing a Preclinical Model of Extrahepatic Abdominal Surgery in Cirrhosis and Non-cirrhotic Portal Hypertension

**Figure 1:**
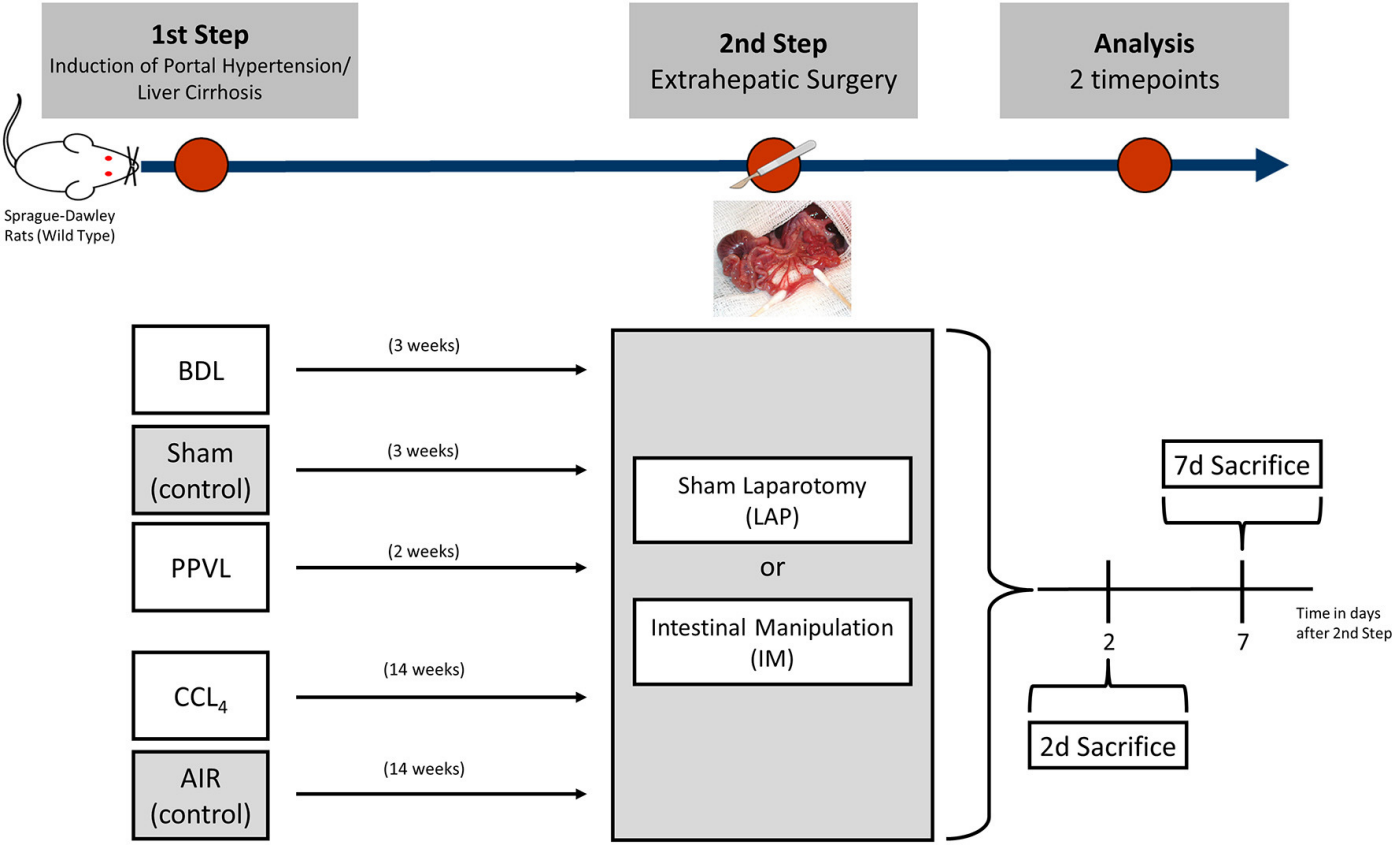
Diagram showing how a two-step pre-clinical model of extrahepatic abdominal surgery (intestinal manipulation) in Sprague-Dawley rats with cirrhosis induced through bile duct ligation (BDL) or inhalation of tetrachlormethan (CCL4) and non-cirrhotic portal hypertension via partial portal vein ligation (PPVL) was established with two timepoints of final analysis.

A two-step animal model of extrahepatic abdominal surgery was established. Cirrhosis was induced via bile duct ligation (BDL) and CCL4-intoxication via inhalation, non-cirrhotic portal hypertension via partial portal vein ligation (PPVL) as previously described (Uschner et al., 2015; Klein et al., 2017). 3 weeks after BDL or PPVL and 14 weeks after CCL4-intoxication (stage of compensated cirrhosis) intestinal manipulation (IM) was performed as previously described (Bortscher et al., 2012; Chang et al., 2012). 2 and 7 days after IM *in-vivo* portal pressure measurement was performed according to established protocol. Animals were then sacrificed and harvested. The experimental design is shown in Figure 1.

### Bile Duct Ligation (BDL)

BDL was performed as previously described in a sterile environment (Uschner et al., 2015; Klein et al., 2017). In short, the common bile duct was ligated twice and dissected between the two ligatures to induce cholestatic cirrhosis. Sham animals received a median laparotomy (group name: Sham). All BDL procedures were performed by the same individual.

### CCL4-Inhalation

Inhalation with CCL4 (abcr, Karlsruhe, Germany) was started at the age of 4 weeks (80–100 g body weight) and performed as previously described (Klein et al., 2017; Brol et al., 2019). Inhalation was done twice a week in growing intervals of 30 s. Reaching 5 min, animals inhaled CCL4 until week 14 (stage of compensated cirrhosis). All animals received phenobarbital (0.33 g/l) via drinking water for induction of cytochrome P-450 metabolic activity starting from 1 week before CCL4 inhalation until animal sacrifice. Inhalation was stopped 3 days before IM as a model of toxic cirrhosis with removal of the injuring agent. Age-matched animals without CCL4 inhalation served as controls (group name: AIR).

### Partial Portal Vein Ligation (PPVL)

To induce non-cirrhotic portal hypertension via PPVL, the portal vein was ligated around a 22 G needle. After ligation, the 22 G needle was removed immediately, resulting in a smaller diameter of the portal vein with consecutive development of non-cirrhotic portal hypertension. The same sham group used for the BDL group served as controls (group name: Sham). All PPVL procedures were performed by the same individual.

### Intestinal Manipulation (Model of Extrahepatic Abdominal Surgery)

Intestinal manipulation (IM) was performed as previously described (Chang et al., 2012). IM was chosen as an established standardized model associated with postsurgical local inflammation and breakup of extracellular matrix in the gut wall (Chang et al., 2012). After median laparotomy, cecum and small bowel were placed on moist gauze outside the abdominal cavity. Then the entire small bowel and colon were manipulated between two sterile cotton swabs twice in a standardized fashion. The intestine was kept moist with saline at all times. After IM the intestine was placed back into the abdominal cavity, the abdomen was then closed with two layers of sutures. Age-matched animals only receiving a median laparotomy without IM served as sham controls (group name: LAP). All IM procedures were performed by the same individual.

### Portal Pressure Measurement and Animal Sacrifice

Before sacrifice animals were put into anaethesia with an intraperitoneal injection of ketamine/xylazin (dose: ketamin 100 mg/kg/body weight (bw)/xylazin: 20 mg/kg/bw). After median laparotomy, for *in-vivo* portal pressure measurements the portal vein was dissected and punctured with a polyethylen catheter (B. Braun, Melsungen, Germany). The catheter was fixated with a vascular clamp. Portal pressure was then recorded over a time of 5 min under echocardiogram monitoring using PowerLab 8/35 and LabChart Software (ADInstruments, Dunedin, New Zealand). The used value of portal pressure and heart frequency of one biological replicate for further analysis, was determined as mean of three randomly chosen values in the recorded phase after calibrated recording. After portal pressure measurement and aquiring blood samples from the caval vein, the animal was sacrificed by dissecting the inferior caval vein. Liver samples were snap frozen in liquid nitrogen at −80 °C, fixated in 4% paraformaldehyde and embedded in paraffin or in Tissue Tek OCT (Sakura Finetek, Staufen, Germany).

### Measurement of Hydroxproline Content in Liver Samples

Hydroxyproline content measurement was performed as described previously (Brol et al., 2019). Snap-frozen liver samples were weighed and dissolved in 12 N hydrochloric acid at 110 °C, then homogenized and incubated for another 16 h at 110 °C. After filtering, samples were dissolved in methanol and oxidized in a chloramine T buffer. Finally, Ehrlich's reagent was added, the photometric product was measured at 558 nm wave length.

### Parameters of Hepatic Inflammation and Circulating Endotoxin Levels

Hepatic inflammation was assessed by mRNA gene expression. RNA isolation was done using the ReliaPrep RNA Miniprep Systems (Promega, Madison, WI). For cDNA synthesis ImProm-II Reverse Transcription System (Promega, Madison, WI) was used. For every sample, two DNase digestion steps were done for genomic DNA to be disposed. Quantitative PCR (qPCR) was carried out using TaqMan gene expression assays (Thermo Fisher Scientific, Waltham, MA) according to the manufacturer's protocol. qPCR amplification was performed on the 7300 Real-Time PCR Cycler (Applied Biosystems, Foster City, CA). qPCR analyses were done using duplicates. Gene expression was calculated with the Delta-Delta CT method. 18s rRNA was used as housekeeping gene. Levels of gene expression are shown as x-fold compared to the respective control group. A list of gene expression assays is shown in Supplementary Table 1.

Circulating endotoxin levels were measured using the Pierce Limulus Amebocyte Lysate (LAL) Chromogenic Endotoxin Quantitation Kit (Thermo Fisher Scientific, Oberhausen, Germany) according to the manufacturer's protocol. In short, all samples were diluted and adjusted to a pH between 6 and 8. After pipetting standards and samples on a 96-well plate and incubating at 37 °C for 4 h, activation in the modified LAL was stopped. Endotoxin concentration was then photometrically measured at 405 nm wavelength. Levels of endotoxin are expressed as EU/ml. Only endotoxin-free plastic ware or sterile glass ware was used for the experiment.

### Histological Staining and Quantification

Sirius red and Hematoxylin and eosin (HE) stainings were performed on paraffin slides (2–3 μm) of the liver as previously described (Trebicka et al., 2011; Schierwagen et al., 2015; Brol et al., 2019). Stainings were captured with a Nikon Digital Sight DS-Vi1 microscope (Chiyoda, Tokyo, Japan) and quantified via ImageJ software (V.1.51q; National Institutes of Health, Bethesda, USA) using macros for automatized quantification and color detection. Individual samples were controlled for correct analysis, if color analysis was not executed properly, threshold was adjusted manually. Images were taken in 10-fold magnification and a minimum of 10 representative fields per biological replicate were taken into analysis.

### Analysis of Laboratory Parameters of Liver Function

Electrolytes (sodium, potassium) and parameters of liver function (alkaline phosphatase (ALP), alanine aminotransferase (ALT), aspartate aminotransferase (AST), albumin (ALB), total protein, and ammonia were analyzed in serum using the Cobas 8000 (Roche Diagnostics, Rotkreuz, Switzerland), modules 8000 ISE, c502, and c702 according to the manufacturer's protocol.

### Statistical Analyses

Statistical analyses were performed using Prism V.5.0 (GraphPad, San Diego, CA). Data are expressed as means ± SEM. For comparisons between two groups, student's *t*-test was used. *P*-values ≤ 0.05 were considered significant.

## Results

### Establishing a Preclinical Model of Extrahepatic Intestinal Surgery in Different Animal Models of Cirrhotic or Non-cirrhotic Portal Hypertension

Only animals that recovered completely from the first operation (BDL, PPVL, Sham) were included in the final analysis. Animals were then randomized into a group that underwent intestinal manipulation (IM) or median laparotomy (LAP). Animals presenting with ascites as a clinical sign of AD prior to IM or LAP were excluded from the experiment.

Due to the more aggressive nature of cirrhosis and expected higher postoperative mortality rate in BDL, a preliminary study had to be performed to determine the optimal time for IM after induction of cirrhosis. This preliminary study was approved within the applied project. When IM was performed 28 days after BDL, postoperative mortality was high, as expected (30%, data not shown). Therefore, the time point of 21 days (3 weeks) after BDL with a mortality of 10% after IM was established for the experiment.

### Postoperative Mortality and Development of Ascites After Intestinal Manipulation

Combined postoperative mortality for BDL, PPVL, and CCL4 groups after IM for both timepoints (2 and 7 days after IM) was 10, 0, and 6%, respectively. Due to a low number of animal deaths without significant distribution concerning time after IM or operation type (IM/LAP), survival analysis was not performed. No deaths were recorded in all control groups (Sham (controls for BDL and PPVL), AIR (controls for CCL4) (Figure 1) after IM or LAP. In the BDL group that underwent IM and were sacrificed 7 days after IM, 3 (43%) developed ascites, vs. 1 (13%) animal in the control group (*p* = 0.3). The rate of development of ascites in the CCL4 group was similar 7 days after IM (IM 3 (38%) vs. LAP 1 (14%), *p* = 0.5). No animals in in BDL and CCL4 groups developed ascites 2 days after IM, also none of the animals belonging to the PPVL groups developed ascites.

### Weight Development

Significant weight differences were observed in all models of cirrhosis or non-cirrhotic portal hypertension at time of IM, compared to the respective control groups (Sham, Air), with a significantly lower body weight in the PPVL, BDL, and CCL4 groups at the time of IM vs. LAP (Supplementary Figure 1).

Moreover, significant weight loss was observed in some groups after IM vs. LAP. In the BDL model, 7 days after IM animals lost significantly more weight compared to LAP, but not at 2 days (**Table 1B**). The same effect was observed in the CCL4 groups (**Table 1D**). In the control and PPVL groups, weight loss before and after IM, was not statistically significant (**Tables 1B**,**D**,**F**).

### Laboratory Parameters of Liver Function at the Time of Sacrifice

Among laboratory parameters representing liver function in the Sham and AIR groups, no significant changes could be observed between IM and LAP groups (Table 1A).

**Table 1A:** General characteristics and clinical data of rats undergoing Sham/BDL at sacrifice 2 days after IM/LAP.

**Parameter**		**Sham + LAP *n* = 8**	**Sham + IM *n* = 8**	***p***	**BDL + LAP *n* = 7**	**BDL + IM *n* = 7**	***p***
Weight data	Liver weight [g]	15 ± 0.6	14.4 ± 0.8	0.41	13.7 ± 0.8	13.3 ± 0.8	0.7
	Body weight at sacrifice [g]	394.6 ± 8.5	393.2 ± 17.9	0.94	330.9 ± 18.2^#^	329 ± 15.3^#^	0.94
	Weight development surgery - sacrifice [%]	−0.25 ± 1.3	−2.7 ± 3.2	0.52	−5.3 ± 2.2^$^	−5.4 ± 0.9^$^	0.94
Baseline laboratory	Sodium [mmol/l]	134.8 ± 2.6	137.0 ± 1.2	0.41	137.4 ± 1.2	139 ± 1.1	0.39
	ALP [U/L]	148.2 ± 21.3	141.3 ± 10.4	0.76	131.3 ± 8.3^$^	151.8 ± 29.3^$^	0.48
	AST [U/L]	88.8 ± 15.7	81.3 ± 6.3	0.63	96 ± 4.7^$^	110.8 ± 17.3^$^	0.39
	ALT [U/L]	47.3 ± 4	38.7 ± 3.4	0.15	47.3 ± 3.5^$^	47.3 ± 3.5^$^	0.47
	ALB [g/L]	30.4 ± 0.5	31.7 ± 1.3	0.41	30.6 ± 0.5^$^	27.3 ± 0.9^#^	0.009**
	TP [g/L]	50.3 ± 0.6	51.9 ± 1.8	0.49	50 ± 0.6^$^	47.3 ± 1.3^$^	0.07
	Urea [mg/dl]	38.0 ± 2.4	44.3 ± 6.0	0.41	37.8 ± 2.5^$^	42.0 ± 4.9^$^	0.44
	Ammonia [μmol/L]	121.3 ± 6.4	106.7 ± 8.1	0.2	101.6 ± 18.5^$^	191.5 ± 17.6^#^	0.007**
Hemodynamics	Portal Pressure [mmHg]	4.8 ± 0.5	5.1 ± 0.5	0.64	10.8 ± 0.5^##^	13.9 ± 0.8^###^	0.011^*^
	Heart frequency [bpm]	195 ± 12.8	200 ± 12.8	0.77	202.2 ± 22.0	208.9 ± 13.9	0.82

*Sham vs. BDL*:

# *p ≤ 0.05*;

## *p ≤ 0.01*;

### *p ≤ 0.001*;

$ *ns. (p > 0.05). BDL + LAP vs. BDL + IM*:

* *p ≤ 0.05*.

In the BDL groups, Alkaline phosphatase (ALP) and Aspartate aminotransferase (AST) were significantly elevated 7 days after IM vs. LAP (Table 1A). Albumin levels were significantly lower, 2 and 7 days after IM vs. LAP (Table 1A). Interestingly, ammonia serum levels were elevated 2 days after IM vs. LAP (Table 1B).

**Table 1B:** General characteristics and clinical data of rats undergoing Sham/BDL at sacrifice 7 days after IM/LAP.

**Parameter**		**Sham + LAP *n* = 8**	**Sham + IM *n* = 8**	***p***	**BDL + LAP *n* = 8**	**BDL + IM *n* = 7**	***p***
Weight data	Liver weight [g]	17.2 ± 0.4	15.9 ± 0.8	0.17	12.8 ± 1.3	15.3 ± 1.4	0.24
	Body weight at sacrifice [g]	408.5 ± 7.3	396.3 ± 12.6	0.42	345.0 ± 34.4^$^	312.6 ± 16.6^##^	0.42
	Weight development surgery - sacrifice [%]	−1.1 ± 1	−1.4 ± 1.8	0.9	4.5 ± 4^$^	−8.4 ± 3^$^	0.03^*^
Baseline laboratory	Sodium [mmol/l]	135.9 ± 1.1	137.4 ± 0.6	0.31	141.4 ± 1.4	141.8 ± 0.5	0.83
	ALP [U/L]	160.0 ± 7.1	147.8 ± 11.6	0.36	154 ± 19.7^$^	340.8 ± 57.4^#^	0.01^*^
	AST [U/L]	76.3 ± 6.4	81.5 ± 4	0.48	116.2 ± 22.7^$^	288.3 ± 80.4^#^	0.05^*^
	ALT [U/L]	49.4 ± 3.5	45.2 ± 1.6	0.36	50.2 ± 11.7^$^	69.8 ± 13.7^$^	0.31
	ALB [g/L]	32.6 ± 0.6	32.7 ± 0.9	0.92	32.7 ± 1.6^$^	22.3 ± 2.1^##^	0.002**
	TP [g/L]	52.1 ± 0.6	50.3 ± 1.4	0.22	50.5 ± 1.7^$^	42.5 ± 2.7^##^	0.01^*^
	Urea [mg/dl]	36.7 ± 3.6	31.3 ± 1.8	0.25	29.5 ± 2.4^$^	44.1 ± 5.7^$^	0.07
	Ammonia [μmol/L]	92.2 ± 14.4	80.3 ± 19.11	0.63	100 ± 12.9^$^	223.6 ± 65^$^	0.07
Hemodynamics	Portal Pressure [mmHg]	4.5 ± 0.6	4.4 ± 0.2	0.82	10.0 ± 0.7^###^	12.4 ± 0.6^###^	0.02^*^
	Heart frequency [bpm]	205.8 ± 16.7	196.6 ± 14.2	0.73	199.1 ± 20.34	197.2 ± 15.37	0.94

*Sham vs. BDL*:

^#^*p ≤ 0.05*;

## *p ≤ 0.01*;

### *p ≤ 0.001*;

$ *ns. (p > 0.05). BDL + LAP vs. BDL + IM*:

* *p ≤ 0.05*.

In the CCL4 group, AST and ALT were significantly elevated 7 days after IM vs. LAP (Table 1D), while albumin levels were significantly lower (Table 1D). No significant differences in laboratory parameters were observed 2 days after IM vs. LAP.

**Table 1C:** General characteristics and clinical data of rats receiving AIR/CCL4 at sacrifice 2 days after IM/LAP.

**Parameter**		**Air + LAP *n* = 8**	**Air + IM *n* = 8**	***p***	**CCL4 + LAP *n* = 7**	**CCL4 + IM *n* = 8**	***p***
Weight data	Liver weight [g]	19.6 ± 0.6	18.9 ± 0.7	0.45	19.2 ± 0.7	17.6 ± 0.6	0.1
	Body weight at sacrifice [g]	546.5 ± 11.7	546.8 ± 11.4	0.99	438.4 ± 15.12^###^	422.8 ± 22.58^##^	0.57
	Weight development surgery - sacrifice [%]	−3.8 ± 0.6	−6.1 ± 1.0	0.1	−1.6 ± 2.0^$^	−7.1 ± 1.3^$^	0.07
Baseline laboratory	Sodium [mmol/l]	139.4 ± 0.2	138.2 ± 1.9	0.6	142.2 ± 0.3	142.5 ± 1.0	0.8
	ALP [U/L]	95.7 ± 10.7	79.6 ± 7.2	0.3	177.4 ± 20^#^	150 ± 27.4^#^	0.4
	AST [U/L]	94.8 ± 10.9	102.4 ± 7.0	0.6	338.3 ± 29.2^###^	405 ± 77.7^#^	0.5
	ALT [U/L]	42.8 ± 1.2	36.6 ± 3.5	0.13	132.4 ± 16.7^###^	131.2 ± 22.7^##^	0.96
	ALB [g/L]	33.5 ± 0.9	35.0 ± 1.2	0.4	33.3 ± 0.9^$^	32.5 ± 0.8^$^	0.6
	TP [g/L]	51.4 ± 1.8	53.4 ± 2.0	0.5	49.5 ± 1.3^$^	50.2 ± 1.4^$^	0.7
	Urea [mg/dl]	36.4 ± 1.2	39.9 ± 2.0	0.2	25.2 ± 2.6^##^	29.6 ± 3.3^#^	0.3
	Ammonia [μmol/L]	73.8 ± 5.3	106.8 ± 26	0.24	107.6 ± 17.9^##^	85.8 ± 6.0^###^	0.3
Hemodynamics	Portal Pressure [mmHg]	4.8 ± 0.6	5.0 ± 0.7	0.81	10.7 ± 0.3^###^	13.3 ± 0.8^###^	0.03^*^
	Heart frequency [bpm]	205.9 ± 11.03	201.0 ± 14.6	0.8	203.7 ± 15.0	197.5 ± 34.8	0.86

*AIR vs. CCL4*:

# *p ≤ 0.05*;

## *p ≤ 0.01*;

### *p ≤ 0.001*;

$ *ns. (p > 0.05). BDL + LAP vs. BDL + IM*:

* *p ≤ 0.05*.

**Table 1D:** General characteristics and clinical data of rats receiving AIR/CCL4 at sacrifice 7 days after IM/LAP.

**Parameter**		**Air + LAP *n* = 8**	**Air + IM *n* = 8**	***p***	**CCL4 + LAP *n* = 8**	**CCL4 + IM *n* = 7**	***p***
Weight data	Liver weight [g]	18.1 ± 0.9	18.8 ± 0.6	0.7	17.7 ± 1.1	16.2 ± 0.9	0.3
	Body weight at sacrifice [g]	540.7 ± 14.8	553.0 ± 14.1	0.6	465.6 ± 4.1^##^	404.6 ± 9.7^###^	<0.001^***^
	Weight development surgery - sacrifice [%]	−4.0 ± 0.5	−3.9 ± 0.7	0.9	−1.2 ± 0.3^$^	−10.7 ± 2.2^$^	0.005^**^
Baseline laboratory	Sodium [mmol/l]	138.8 ± 0.3	139.8 ± 0.6	0.2	141.6 ± 0.8	141.5 ± 1.3	1
	ALP [U/L]	79.2± 11.6	77.4 ± 2.8	0.9	146 ± 23^#^	194.1 ± 22.6^##^	0.17
	AST [U/L]	75.8 ± 5.7	103. ± 12.1	0.08	171.0 ± 29.0^#^	306.7 ± 30.7^###^	0.02^*^
	ALT [U/L]	38.0 ± 2.1	43.8 ± 3.7	0.21	77.4 ± 5.4^###^	118.7 ± 6.0^###^	<0.001^***^
	ALB [g/L]	34.2 ± 1.0	33.2 ± 0.6	0.4	34.6 ± 1.2^$^	28.0 ± 1.0^##^	0.0017^**^
	TP [g/L]	47.7 ± 1.4	48.0 ± 1.5	0.9	49.1 ± 1.1^$^	45.4 ± 1.3^$^	0.1
	Urea [mg/dl]	31.9 ± 0.9	40.3 ± 3.3	0.02^*^	25.8 ± 2.1^#^	30.7 ± 1.9^#^	0.1
	Ammonia [μmol/L]	100.2 ± 18.7	109.4 ± 38.4	0.8	86.8 ± 18.3^#^	111.75 ± 27^$^	0.5
Hemodynamics	Portal Pressure [mmHg]	4.7 ± 0.7	4.4 ± 0.3	0.71	10.2 ± 0.6^###^	13.7 ± 1.1^###^	0.02^*^
	Heart frequency [bpm]	210.2 ± 27.94	194.0 ± 0.5	0.59	190.8 ± 8.3	191.2 ± 8.6	0.43

*AIR vs. CCL4*:

# *p ≤ 0.05*;

## *p ≤ 0.01*;

### *p ≤ 0.001*;

$ *ns. (p > 0.05). CCL4 + LAP vs. CCL4 + IM*:

* *p ≤ 0.05*;

** *p ≤ 0.01*;

*** *p ≤ 0.001*.

In the PPVL group 2 days after IM, AST was significantly elevated vs. LAP (Table 1E). No other significant differences were observed 2 and 7 days after IM vs. LAP.

**Table 1E:** General characteristics and clinical data of rats undergoing Sham/PPVL at sacrifice 2 days after IM/LAP.

**Parameter**		**Sham + LAP *n* =8**	**Sham + IM *n* = 8**	***p***	**PPVL + LAP *n* = 8**	**PPVL + IM *n* = 8**	***p***
Weight data	Liver weight [g]	15 ± 0.6	14.4 ± 0.8	0.41	14.1 ± 0.7	15.4 ± 1.0	0.34
	Body weight at sacrifice [g]	394.6 ± 8.5	393.2 ± 17.9	0.94	388.1 ± 11.1^$^	385.7 ± 9.1^$^	0.87
	Weight development surgery - sacrifice [%]	−0.25 ± 1.3	−2.7 ± 3.2	0.52	0.8 ± 1.0^$^	−1.8 ± 0.5^$^	0.16
Baseline laboratory	Sodium [mmol/l]	134.8 ± 2.6	137.0 ± 1.2	0.41	140.1 ± 1.0	139.9 ± 0.6	0.85
	ALP [U/L]	148.2 ± 21.3	141.3 ± 10.4	0.76	132.1 ± 8.3^$^	135.5 ± 5.6^$^	0.73
	AST [U/L]	88.8 ± 15.7	81.3 ± 6.3	0.63	82.0 ± 6.0^$^	123 ± 16.6^$^	0.04^*^
	ALT [U/L]	47.3 ± 4	38.7 ± 3.4	0.15	57.4 ± 3.1^$^	60.2 ± 4.0^$^	0.6
	ALB [g/L]	30.4 ± 0.5	31.7 ± 1.3	0.41	31.7 ± 0.8^$^	31.6 ± 0.6^$^	0.96
	TP [g/L]	50.3 ± 0.6	51.9 ± 1.8	0.49	48.6 ± 0.9^$^	48.8 ± 1.0^$^	0.85
	Urea [mg/dl]	38.0 ± 2.4	44.3 ± 6.0	0.41	41 ± 2.4^$^	36.3 ± 1.4^$^	0.1
	Ammonia [μmol/L]	121.3 ± 6.4	106.7 ± 8.1	0.2	138.8 ± 15.1^$^	136.2 ± 13.8^$^	0.91
Hemodynamics	Portal Pressure [mmHg]	4.8 ± 0.5	5.1 ± 0.5	0.64	13.3 ± 0.9^###^	15.8 ± 1.0^###^	0.11
	Heart frequency [bpm]	195 ± 12.8	200 ± 12.8	0.77	211.1 ± 12.2	207.3 ± 14.6	0.84

*Sham vs. PPVL*:

# *p ≤ 0.05*;

## *p ≤ 0.01*;

### *p ≤ 0.001*;

$ *ns. (p > 0.05). CCL4 + LAP vs. CCL4 + IM*:

* *p ≤ 0.05*.

### Development of Hepatic Fibrosis After Intestinal Manipulation

**Figure 2:**
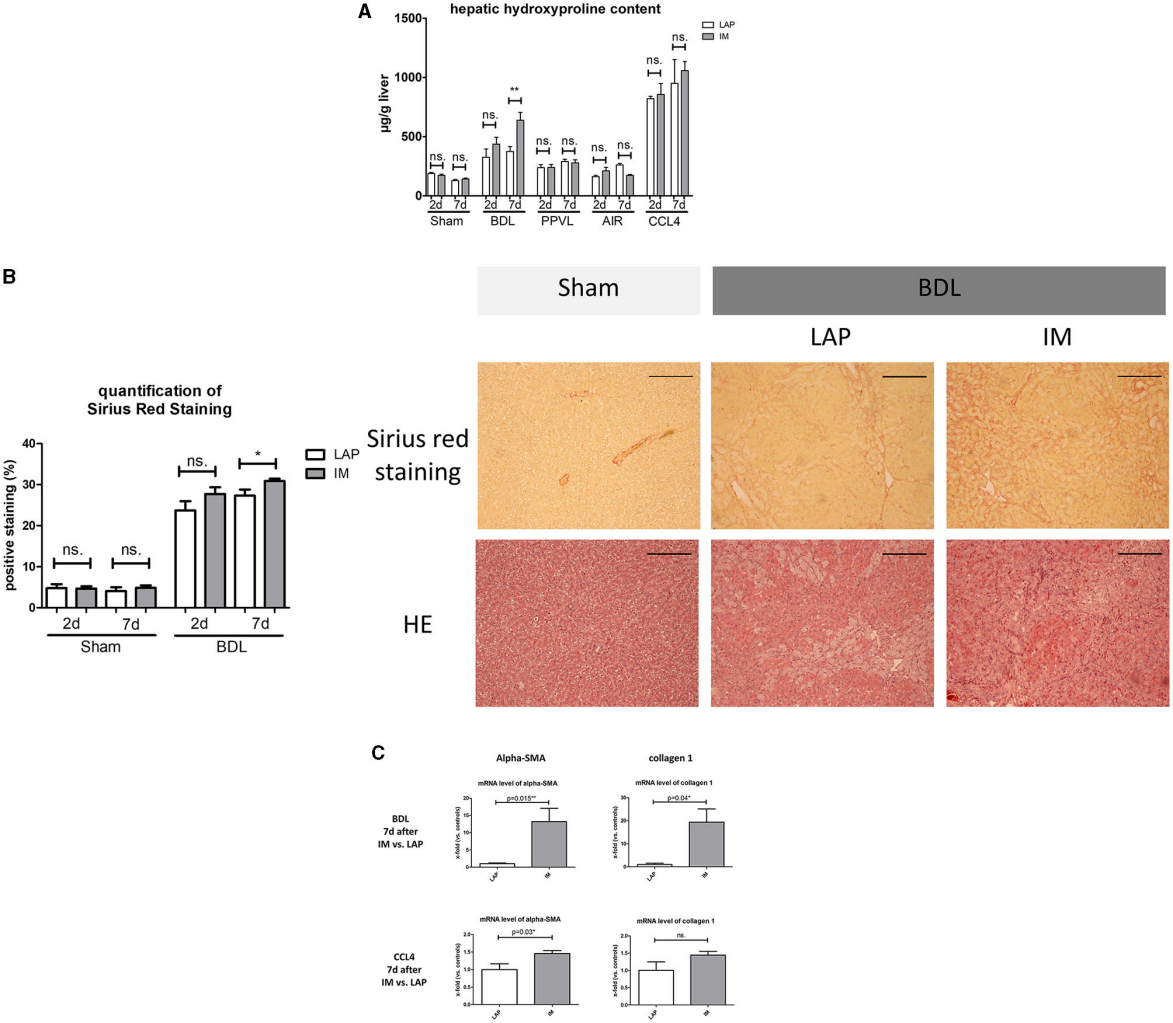
**(A)** Hepatic hydroxyproline content measurements 2 or 7 days after IM vs. LAP, showing progression of fibrosis through a highly significant elevation of hydroxyproline concentration (expressed in μg/g liver) of the liver in the BDL group 7 days after IM vs. LAP. No differences are seen within the Sham, PPVL, and CCL4 groups. **(B)** Representative images of liver samples with Sirius-red staining for assessment of fibrosis with observed significant differences of fibrosis (expressed as percentage of positive stained area) 7 days after intestinal manipulation vs. the respective control group (LAP). Images of Hematoxylin Eosin staining are shown for morphologic comparison. The scale bar is 200 μm. **(C)** mRNA gene expression for alpha-SMA and collagen 1, showing a significant increase of both parameters of fibrosis progression in the BDL group 7 days after IM and for alpha-SMA in the CCL4 group 7 days after IM. Levels of gene expression are shown as x-fold compared to the respective control group. *p ≤ 0.05; **p ≤ 0.01; ns. (p > 0.05).

Hydroxyproline measurement showed significant increase of hydroxyproline levels and significantly larger Sirius-Red stained areas in the BDL group 7 days after IM vs. LAP (Figures 2A,B). In the BDL group 7 days after IM, mRNA expression of alpha-SMA (a marker of profibrogenic hepatic stellate cells) and collagen 1 were significantly upregulated compared to LAP (13- and 19-fold, *p* = 0.02 and *p* = 0.04, respectively) (Figure 2C). In the other cirrhotic and non-cirrhotic groups (PPVL, Sham, Air, CCL4), no significant differences in fibrosis development between animals receiving IM vs. LAP could be observed, except a slight, but significant upregulation of alpha-SMA gene expression in the CCL4 group 7 days after IM compared to LAP (Figures 2A,C; Supplementary Figure 2).

### Portal Pressure After Intestinal Manipulation

**Figure 3:**
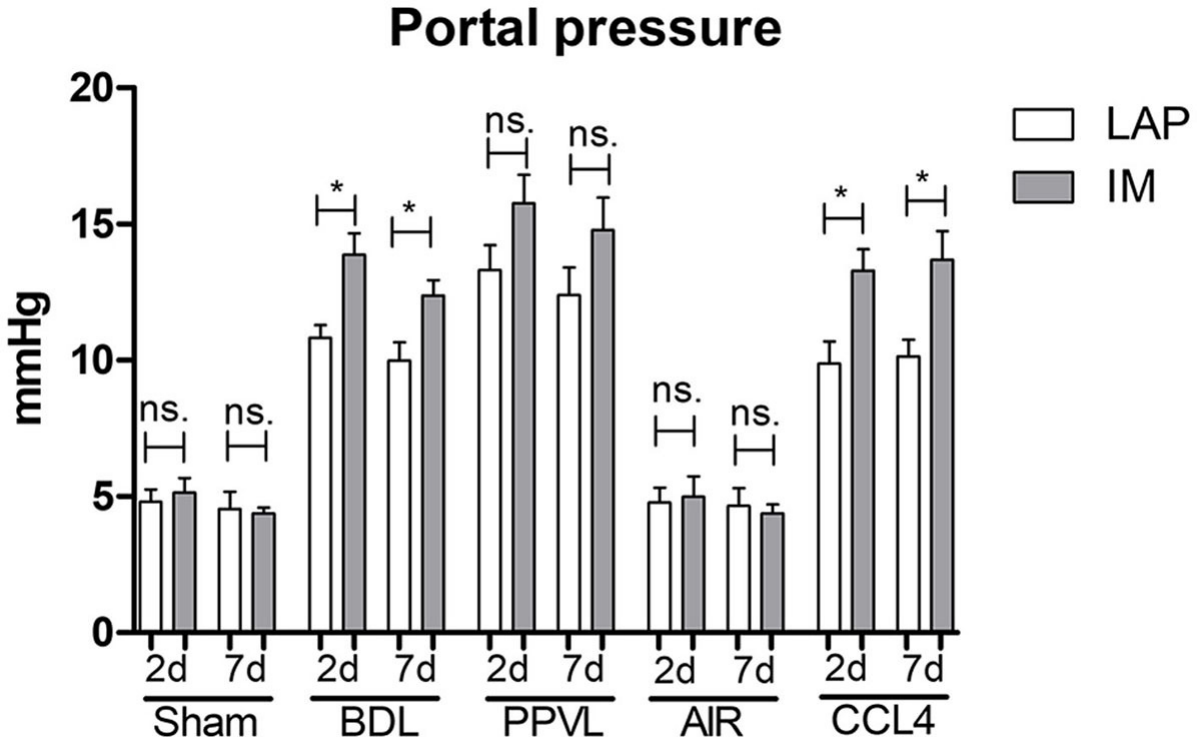
*In-vivo* portal pressure development 2 or 7 days after IM vs. LAP in the different animal models (BDL and PPVL vs. Sham and CCL4 vs. AIR), showing significantly elevated portal pressure after IM in all BDL and CCL4 groups. *p ≤ 0.05; ns. (p > 0.05).

In the cirrhotic groups (BDL and CCL4) portal pressure was significantly elevated 2 and 7 days after IM compared to LAP (BDL 2 d: IM 13.9 ± 0.8 mmHg vs. LAP 10.8 ± 0.5 mmHg, *p* = 0.01^*^; BDL 7 d: IM 12.4 ± 0.6 mmHg vs. LAP 10.0 ± 0.7 mmHg, *p* = 0.02; CCL4 2 d: IM 13.3 ± 0.8 mmHg vs. LAP 10.7 ± 0.3 mmHg, *p* = 0.03; CCL4 7 d: IM 13.7 ± 1.1 mmHg vs. LAP 10.2 ± 0.6 mmHg, *p* = 0.02) (Figure 3; Tables 1A–D). The control groups (Sham, AIR) did not show any significant increase of portal pressure after IM or LAP (Figure 3; Tables 1A–D). While there was a trend of higher portal pressure after IM in the PPVL group, results were not significantly different compared to LAP (Figure 3; Tables 1E,F).

**Table 1F:** General characteristics and clinical data of rats undergoing Sham/PPVL at sacrifice 7 days after IM/LAP.

**Parameter**		**Sham + LAP *n* = 8**	**Sham + IM *n* = 8**	***p***	**PPVL + LAP *n* = 8**	**PPVL + IM *n* = 8**	***p***
Weight data	Liver weight [g]	17.2 ± 0.4	15.9 ± 0.8	0.17	14.2 ± 0.3	13.9 ± 0.4	0.58
	Body weight at sacrifice [g]	408.5 ± 7.3	396.3 ± 12.6	0.42	378.6 ± 8.5^$^	369.3 ± 7.7^$^	0.42
	Weight development surgery - sacrifice [%]	−1.1 ± 1	−1.4 ± 1.8	0.9	−0.3 ± 1.71^$^	−2.2 ± 1.5^$^	0.41
Baseline laboratory	Sodium [mmol/l]	135.9 ± 1.1	137.4 ± 0.6	0.31	139.0 ± 1.1^$^	140.7 ± 0.5^$^	0.2
	ALP [U/L]	160.0 ± 7.1	147.8 ± 11.6	0.36	127.3 ± 7.8^#^	139.7 ± 11.4^$^	0.42
	AST [U/L]	76.3 ± 6.4	81.5 ± 4	0.48	71.9 ± 4.8^$^	79.5 ± 7.3^$^	0.42
	ALT [U/L]	49.4 ± 3.5	45.2 ± 1.6	0.36	48.7 ± 1.4^$^	40.7 ± 2.7^#^	0.11
	ALB [g/L]	32.6 ± 0.6	32.7 ± 0.9	0.92	31.7 ± 0.6^$^	30.0 ± 1.0^$^	0.17
	TP [g/L]	52.1 ± 0.6	50.3 ± 1.4	0.22	50.2 ± 0.7^#^	46.6 ± 2.2^$^	0.15
	Urea [mg/dl]	36.7 ± 3.6	31.3 ± 1.8	0.25	37.8 ± 2.1^$^	34.2 ± 2.1^$^	0.23
	Ammonia [μmol/L]	92.2 ± 14.4	80.3 ± 19.11	0.63	89.5 ± 16^$^	151.7 ± 46.3^#^	0.18
Hemodynamics	Portal Pressure [mmHg]	4.5 ± 0.6	4.4 ± 0.2	0.82	12.4 ± 1.0^###^	14.8 ± 1.2^###^	0.16
	Heart frequency [bpm]	205.8 ± 16.7	196.6 ± 14.2	0.73	222.2 ± 16.9	200.9 ± 2.9	0.1

*Sham vs. PPVL*:

# *p ≤ 0.05*;

### *p ≤ 0.001*;

$ *ns. (p > 0.05)*.

*ALB, albumin; ALP, alkaline phosphatase; ALT, alanine aminotransferase; AST, aspartate aminotransferase; BDL, bile-duct ligation; bpm, beats per minutes; CCL4, tetrachlormethane; IM, intestinal manipulation; LAP, median laparotomy; ns, no significance; PPVL, partial portal vein ligation; TP, total protein*.

### Parameters of Hepatic Inflammation and Circulatory Level of Endotoxins

**Figure 4:**
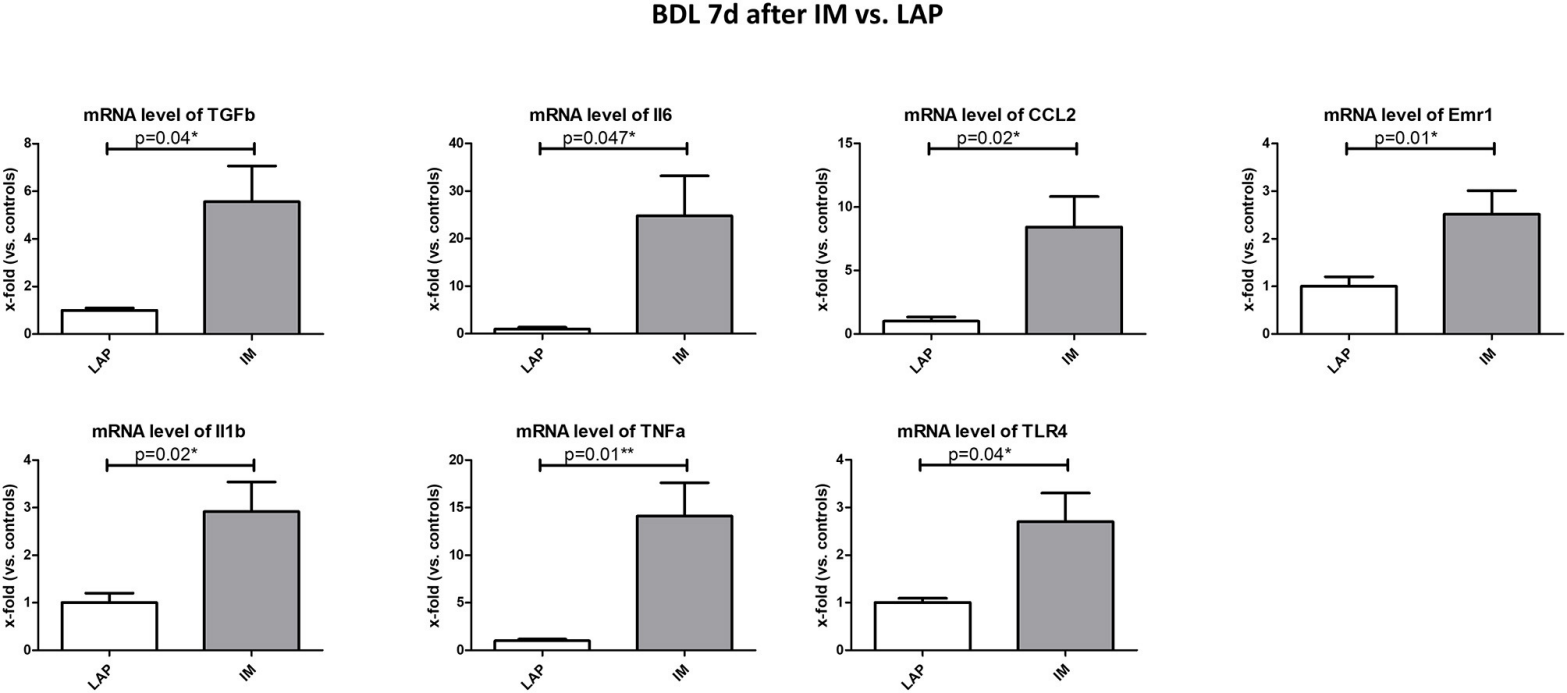
Analysis of parameters of hepatic inflammation (TGFb, IL-6, IL-1b, TNF-alpha, Emr1, TLR-4) in the BDL group 7 days after IM vs. LAP, all significantly elevated. Levels of gene expression are shown as x-fold compared to the respective control group. *p ≤ 0.05; **p ≤ 0.01; ns. (p > 0.05).

Transforming growth factor beta 1 (TGF-beta), interleukin 6 (IL-6), interleukin 1 beta (IL-1b), tumor necrosis factor alpha (TNF-alpha), chemokine (C-C motif) ligand 2 (CCL2), EGF-like module containing mucin-like hormone receptor-like 1 (EMR-1), toll-like receptor 4 (TLR-4) were measured in liver samples via mRNA gene expression as parameters of hepatic inflammation. These inflammatory parameters were elevated in the BDL group 7 days after IM but not 2 days after IM vs. LAP, especially IL-6 (25-fold), TNF-alpha (14-fold) and CCL2 (8-fold) (*p* = 0.05, *p* = 0.006, *p* = 0.02, respectively) (Figure 4). In the CCL4 model IL-6 gene expression was significantly upregulated after IM vs. LAP among the measured inflammatory parameters (Supplementary Figure 3).

Endotoxin levels were measured in the different models 2 and 7 days after IM/LAP as a marker of bacterial translocation as possible trigger for upregulation of hepatic inflammation. No significant differences could be detected 2 and 7 days after IM/LAP between operation type (IM vs. LAP) or blood compartment (portal vein vs. caval vein). However, in both cirrhosis models circulatory level of endotoxins was significantly higher than in the non-cirrhotic groups (Supplementary Figure 4).

## Discussion

This study is the first to characterize portal pressure after abdominal extrahepatic surgery in preclinical models of cirrhosis with ongoing hepatic injury (BDL) and discontinued hepatic injury (CCL4) prior to surgery. It shows that an abdominal extrahepatic surgical procedure significantly increases portal pressure, rendering our model suitable for studying pathomechanisms of post-operative acute decompensation (AD).

Postoperative AD in patients with cirrhosis is a long existing clinical problem which to date limits surgical procedures in cirrhosis. Moreover, AD can precipitate ACLF, resulting in multiorgan-failure and high short-term mortality (Moreau et al., 2013; Trebicka et al., 2020a). In previous studies, our group showed that about 25% of patients with cirrhosis develop post-operative ACLF, including emergency surgery (Klein et al., 2020). Similar rates of post-operative ACLF are demonstrated even in elective surgical procedures, thus establishing surgical procedures as a precipitant of ACLF (Chang et al., 2021). In clinical practice, Child-Turcotte-Pugh-Score is commonly used for pre-operative risk stratification. Higher Child-Turcotte-Pugh-Score at the time of surgery is associated with higher mortality (Friedman, 2010). However, more biomarkers and clinical parameters are needed for better risk stratification for patients with liver cirrhosis in need of a surgical procedure.

Portal pressure seems to play an important role in post-operative outcome of patients with cirrhosis. In a recent prospective study, it was shown that preoperative HVPG below 16 mmHg before a surgical procedure is associated with a better postoperative outcome (Reverter et al., 2019). In a series of smaller studies it was shown that preoperative decompression of portal pressure via transjugular portosystemic shunt (TIPS) improves post-operative outcome, thus the concept of preoperative TIPS has been discussed as well (García-Pagán et al., 2020). However, no studies investigated the evolution of portal pressure after surgery, limiting the investigation of pathophysiological pathways driving post-operative hepatic decompensation. Prospective clinical studies to characterize post-operative measurements of portal pressure in patients with cirrhosis are ethically difficult to perform, given that patients are mostly under postoperative care in the intensive care unit and in danger of AD or ACLF development. Therefore, animal models are needed to explore the mechanisms of postoperative AD or ACLF, and to study the evolution of portal pressure after surgery and its association with potential underlying inflammatory processes.

In our study, we show in two different animal models of cirrhosis (BDL and CCL4), that portal pressure is significantly elevated 2 and 7 days after IM vs. LAP (median laparotomy). Accordingly, in both models, 7 days after IM, there were more clinical events of decompensation such as the development of ascites or significant weight loss. The BDL model seems to mimic the clinical situation after surgery in patients more accurately, showing progression of fibrosis and significant elevation of parameters of hepatic inflammation 7 days after IM and elevation of liver enzymes and ammonia acutely within 2 days after IM. In the BDL model, IM was performed in an earlier stage of fibrosis (3 weeks after BDL), leading to progression of fibrosis and earlier decompensation events after IM vs. LAP. In the CCL4 group however, no significant changes of the fibrosis parameters could be observed after IM compared to LAP. A possible reason might be that IM was performed at more advanced stages of cirrhosis. However, a regression of fibrosis after withdrawal of the injuring agent has been described in the CCL4 model (Nevzorova et al., 2020). In our model CCL4 inhalation was stopped 3 days before IM, which might be masking progression of fibrosis in this model by IM. However, the perioperative discontinuation of the hepatotoxic agents such as alcohol reflects clinical reality. Still, in our CCL4 model IM leads to significantly elevated portal pressure, elevated levels of liver enzymes and alpha-SMA and IL-6 gene expression after IM compared to LAP as relevant surrogate parameters for hepatic inflammation and decompensation.

An elevated portal pressure after surgery may be the expression of increased systemic inflammation. Our data shows significantly upregulated parameters of inflammation after IM, suggesting an association between inflammation and the development of elevated portal pressure after surgery. A close association of HVPG and systemic inflammation has been shown recently (Praktiknjo et al., 2020). A hyperinflammatory state is also a key element of ACLF (Trebicka et al., 2019).

Inflammatory pathways driven by bacterial translocation and mechanisms of sterile inflammation may play a role in post-operative portal pressure elevation. In a recent retrospective study, bowel-related surgery was associated with a poor outcome in patients with cirrhosis, especially in those presenting with ascites and thrombocytopenia (Wetterkamp et al., 2020). Our study supports the role of bacterial translocation, indicated by the significant increase of hepatic TLR-4 expression in the BDL model 7 d after IM and significantly higher levels of endotoxin in the cirrhosis models. However, our data do not show differences of endotoxin levels between IM and LAP groups at 2 and 7 days. It has been shown that major abdominal surgery is associated with transient endotoxemia that peak between 1 and 24 h after surgery (Buttenschoen et al., 2001, 2009). Endotoxin levels might be significantly higher immediately after IM and then quickly decrease after increase of systemic and inflammation markers and upregulation of hepatic inflammation. This hypothesis needs to be further investigated in the future.

It has also been shown that IM leads to a disruption of the gut wall with the release of sterile proinflammatory agents, e.g., extracellular matrix components that lead to local and systemic inflammation (Bortscher et al., 2012; Chang et al., 2012; Nielsen et al., 2015; Lehmann et al., 2019). Our data show significant upregulation of collagen type 1 and elevated hepatic hydroxyproline levels in the BDL model 7 days after IM as expression of fibrosis progression and clinical events of decompensation. A boost of collagens and fragments or neoepitopes of extracellular matrix systemically and in the portal vein have been shown to be significantly associated with outcome in patients with advanced stages of cirrhosis (Leeming et al., 2013, 2015; Nielsen et al., 2015; Praktiknjo et al., 2018a; Lehmann et al., 2019). Our data indicate that extrahepatic bowel surgery, especially in the model of continuous liver injury, may have the same effect, but needs to be confirmed in further studies using this model. We believe that our model is well-suited to study different pathways of inflammation and thus to investigate pathomechanisms of postoperative hepatic decompensation.

Interestingly in the PPVL groups, portal pressure was not significantly elevated after IM but showed the same trend as the cirrhotic groups. No deaths or signs of decompensation after IM in this model were recorded, at best, only transient changes were seen in the expression of liver enzymes. IM in this important non-cirrhotic control group was performed relatively early after PPVL. Patients with portal hypertension without cirrhosis, e.g., with vascular disorders of the liver have better postsurgical prognosis, if they are treated early before the presence of liver decompensation (Elkrief et al., 2019). Our data show distinct post-surgical differences of inflammatory pathways between cirrhosis and non-cirrhotic portal hypertension, which can be further evaluated using this model.

Sarcopenia seems to play a role in these animal models of cirrhotic and non-cirrhotic portal hypertension. Our data show significant weight differences during the time of development of cirrhosis or non-cirrhotic portal hypertension. Weight loss seems to be more significant after IM in models of cirrhosis than in sham and PPVL animals. While weight loss in cirrhosis is a well-known fact, molecular mechanisms are still not fully understood, since obtaining muscle biopsies in patients might be ethically difficult. In recent studies it has been shown that muscle mass in patients with cirrhosis is associated with outcome and ACLF (Praktiknjo et al., 2018b, 2019). Pathophysiological investigation of the role of sarcopenia in the development of AD and ACLF especially after surgery in this model should be performed in the future, but is beyond the scope of this study.

There are several limitations to the study. Different surgical models, especially IM, might be dependent on the animal surgeon. However, to remove bias, all surgical procedures were performed by the same and trained individual for each surgery type (BDL, Sham, IM, LAP). Surgical procedures were performed in a block design, and animals were randomized into the different groups. Clear-cut criteria of AD in animals are missing, but our data includes relevant surrogate parameters of systemic inflammation and important laboratory parameters as well as clinical features of decompensation. Whether progression of fibrosis and inflammation can be seen more clearly in the CCL4 model without the removal of the hepatotoxic agent CCL4 remains to be investigated. Finally, the role and relevance of bacterial translocation and integrity of intestinal barrier need to be assessed in more detail in further experiments including groups treated with antibiotics.

In conclusion, this study showed significantly elevated portal pressure and systemic inflammation in preclinical models of cirrhosis after IM. It also shows progression of fibrosis especially in models of continuous liver injury. These models may be useful to investigate pathophysiological mechanisms of post-operative decompensation. Lowering the risk of postoperative portal pressure elevation may be a therapeutic target.

## Data Availability Statement

The datasets presented in this article are not readily available because restrictions according to GDPR and LANUV (local animal authority) apply. Requests to access the datasets should be directed to Dr. Michael Praktiknjo, michael.praktiknjo@ukbonn.de.

## Ethics Statement

The animal study was reviewed and approved by Landesamt für Natur, Umwelt und Verbraucherschutz Nordrhein-Westfalen (81-02-04.2018.A348).

## Author Contributions

JC, JM, MG, and MH: acquisition of data, analysis, interpretation of data, drafting of the manuscript, and statistical analysis. NB, RD-P, BS-W, and GK: acquisition of data, analysis, and interpretation of data and critical revision of the manuscript regarding important intellectual content. MO, LP, SK, FU, MB, TV, PL, and JK: interpretation of data and critical revision of the manuscript regarding important intellectual content. CJ and CS: administrative, technical and material support, and critical revision of the manuscript regarding important intellectual content. SW, JT, and MP: study concept and design, analysis and interpretation of data, drafting of the manuscript, critical revision of the manuscript regarding important intellectual content, final approval of the version to be published, administrative, technical and material support, and study supervision. All authors contributed to the article and approved the submitted version.

## Conflict of Interest

The authors declare that the research was conducted in the absence of any commercial or financial relationships that could be construed as a potential conflict of interest.

## Publisher's Note

All claims expressed in this article are solely those of the authors and do not necessarily represent those of their affiliated organizations, or those of the publisher, the editors and the reviewers. Any product that may be evaluated in this article, or claim that may be made by its manufacturer, is not guaranteed or endorsed by the publisher.

## Abbreviations

18s rRNA: Eukaryotic 18S ribosomal ribonucleic acid
ACLF: acute-on-chronic liver failure
AD: acute decompensation
ALB: albumin
ALP: alkaline phosphatase
ALT: alanine aminotransferase
AST: aspartate aminotransferase
BDL: bile-duct ligation
CCL2: chemokine (C-C motif) ligand 2
CCL4: tetrachlormethane
cDNA: complementary desoxyribonucleic acid
CTP: Child-Turcotte-Pugh
EMR-1: EGF-like module containing mucin-like hormone receptor-like 1
HE: hematoxylin and eosin
HVPG: hepatic venous pressure gradient
i.p.: intraperitoneal
ICU: intensive care unit
IL-6: interleukin 6
IL1b: interleukin 1 beta
IM: intestinal manipulation
LAP: median laparotomy
mRNA: messenger ribonucleic acid
PE: Polyethylen
qPCR: real-time polymerase chain reaction
TGF-beta: transforming growth factor beta 1
TIPS: transjugular portosystemic shunt
TLR-4: toll-like receptor 4
TNF-alpha: tumor necrosis factor alpha
TP: total protein.
