# Supplementary material for: Extrahepatic Surgery in Cirrhosis Significantly Increases Portal Pressure in Preclinical Animal Models
Source: Front Physiol. 2021 Aug 20;12:720898. doi: 10.3389/fphys.2021.720898 (PMC8418541; doi:10.3389/fphys.2021.720898)
Supplement: Supplementary file 1 [file Table_1.docx]

**SUPPLEMENTARY TABLE 1.** Taqman assays of nucleotide sequences of the rat (Applied Biosystems, Germany)

| Assay ID | Gene symbol | Gene Name | Reference Sequence | Amplicon Length |
| --- | --- | --- | --- | --- |
| Hs99999901_s1 | 18s-rRNA | Eukaryotic 18S rRNA | X03205.1 | 187 |
| Rn01759928_g1 | Acta2 | Alpha-Smooth Muscle Actin | NM_031004.2 | 65 |
| Rn00580555_m1 | Ccl2 | chemokine (C-C motif) ligand 2 | NM_031530.1 | 95 |
| Rn01463848_m1 | Col1a1 | collagen, type I, alpha 1 | NM_053304.1 | 115 |
| Rn01527631_m1 | Emr1 | EGF-like module containing, mucin-like hormone receptor-like 1 | NM_001007557.1 | 100 |
| Rn00580432_m1 | Il1b | interleukin 1 beta | NM_031512.2 | 74 |
| Rn01410330_m1 | Il6 | interleukin 6 | NM_012589.2 | 121 |
| Rn00572010_m1 | Tgfb1 | transforming growth factor, beta 1 | NM_021578.2 | 65 |
| Rn00569848_m1 | Tlr4 | toll-like receptor 4 | NM_019178.1 | 127 |
| Rn99999017_m1 | Tnf | tumor necrosis factor | NP_036807.1 | 108 |
