# Supplementary figures and images for: Extrahepatic Surgery in Cirrhosis Significantly Increases Portal Pressure in Preclinical Animal Models
Source: Front Physiol. 2021 Aug 20;12:720898. doi: 10.3389/fphys.2021.720898 (PMC8418541; doi:10.3389/fphys.2021.720898)

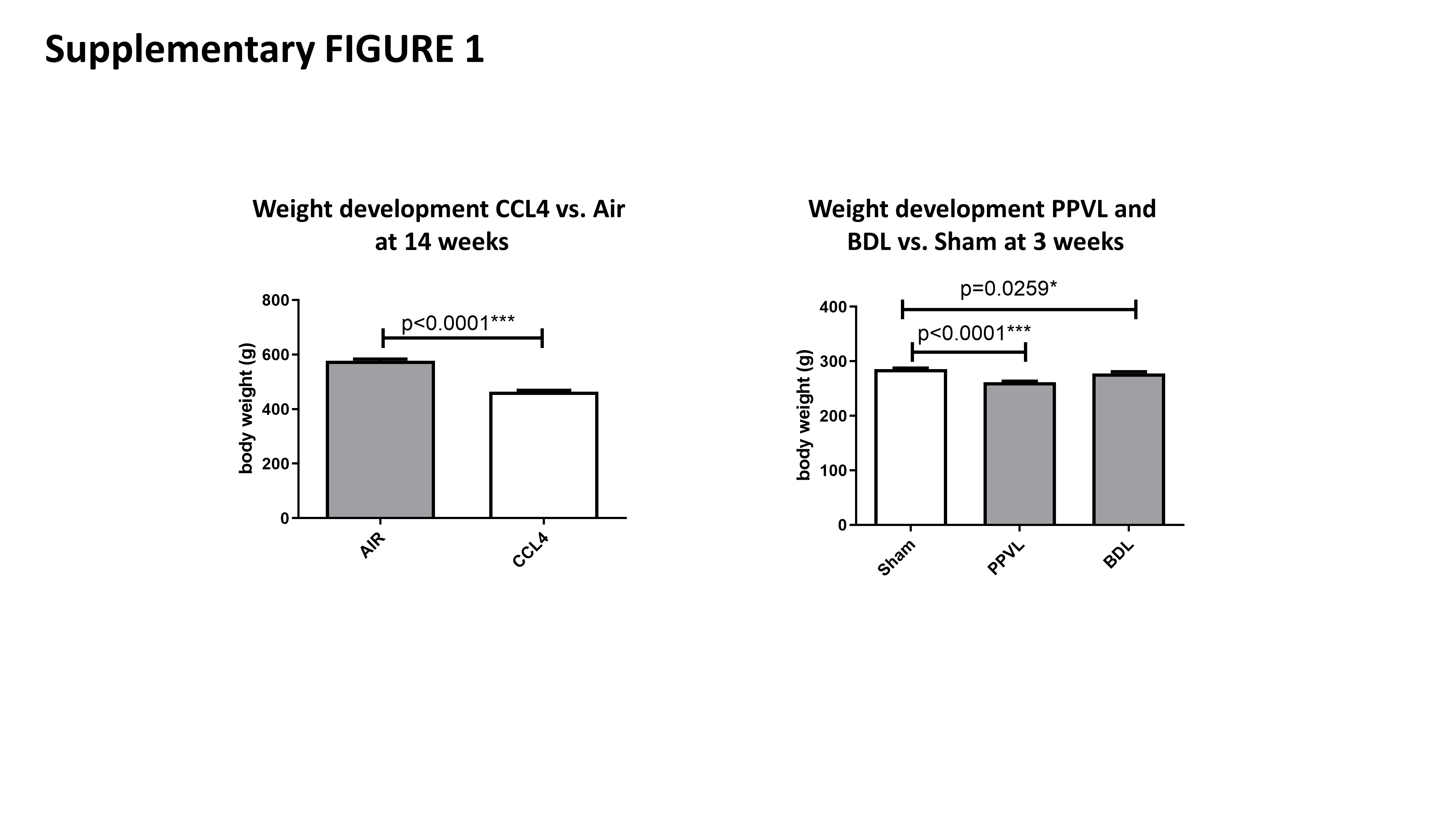

Supplement: Supplementary file 2 [file Image_1.TIF]

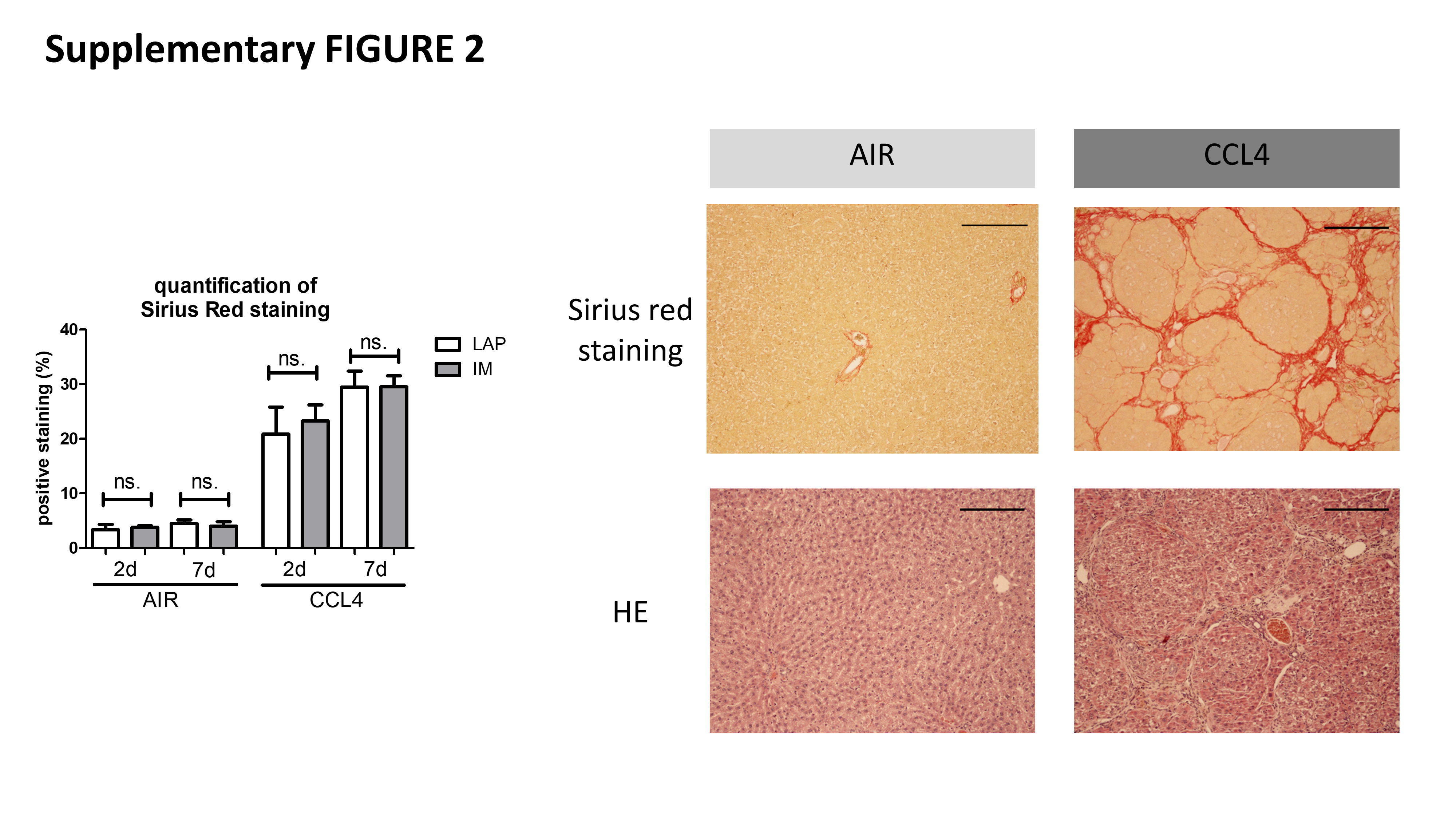

Supplement: Supplementary file 3 [file Image_2.TIF]

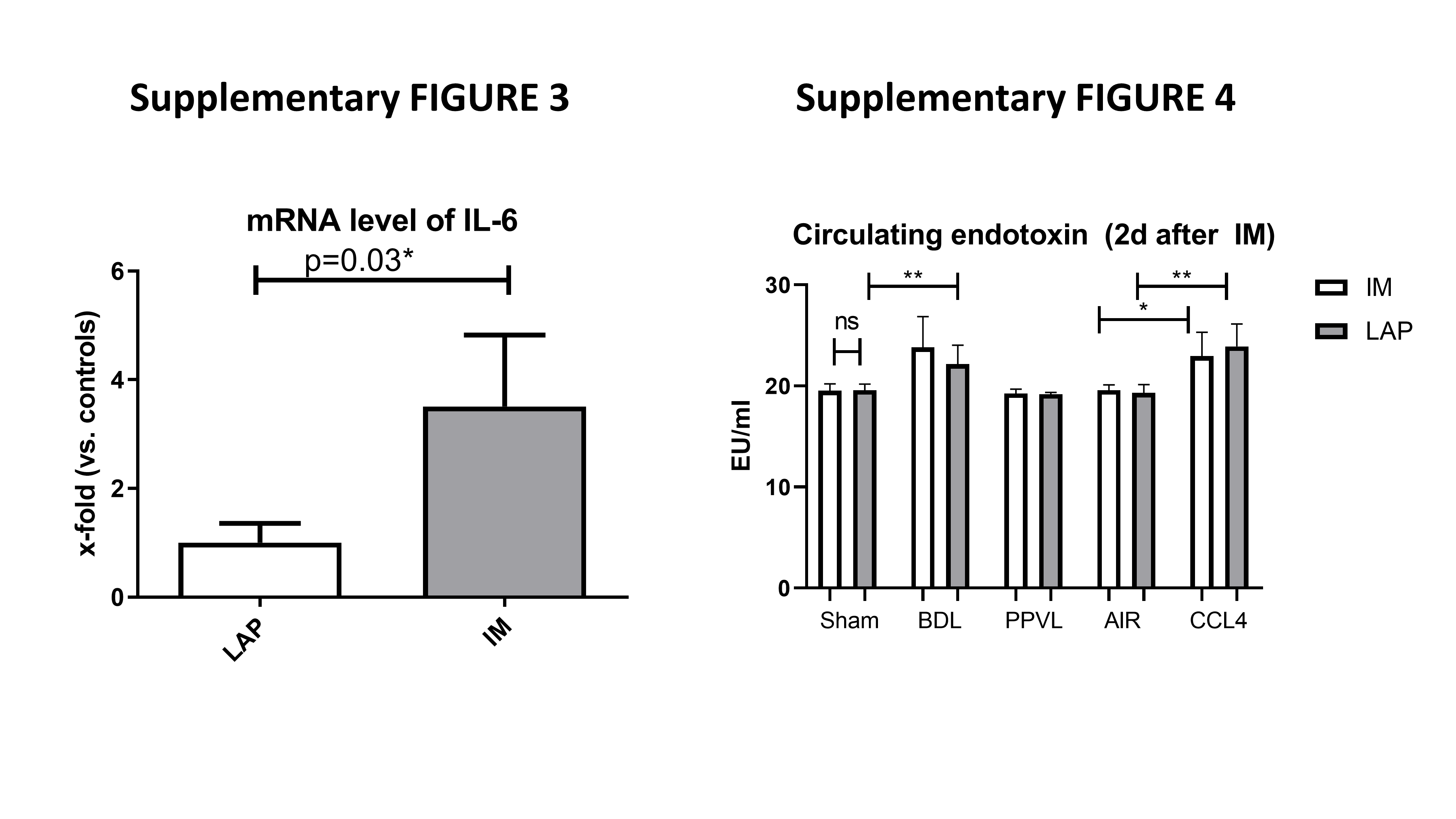

Supplement: Supplementary file 4 [file Image_3.TIF]
