# Supplementary material for: Extrahepatic Surgery in Cirrhosis Significantly Increases Portal Pressure in Preclinical Animal Models
Source: Front Physiol. 2021 Aug 20;12:720898. doi: 10.3389/fphys.2021.720898 (PMC8418541; doi:10.3389/fphys.2021.720898)
Supplement: Supplementary file 5 [file Data_Sheet_1.docx]

**Supplementary Figure 1:**

Weight development between BDL or PPVL vs. Sham and CCL4 vs. AIR groups until the time of intestinal manipulation.

**Supplementary Figure 2:**

Representative images of liver samples with Sirius-red staining for assessment of fibrosis, expressed as percentage of positive stained area, with no differences within CCL4 and AIR groups. Images of Hematoxylin Eosin staining is shown for morphologic comparison. The scale bar is 200 µm.

**Supplementary Figure 3:**

Analysis of hepatic IL-6 gene expression in the CCL4 group 7 days after IM vs. LAP. Levels of gene expression are shown as x-fold compared to the respective control group.

**Supplementary Figure 4:**

Endotoxin levels (representatively for 2 days after IM) measured in serum samples. Endotoxin levels in the cirrhosis groups are significantly higher than in the non-cirrhotic groups. Levels of endotoxin are shown as EU/ml.
